# Implementing automated 3D measurements to quantify reference values and side-to-side differences in the ankle syndesmosis

**DOI:** 10.1038/s41598-023-40599-3

**Published:** 2023-08-23

**Authors:** Matthias Peiffer, Ide Van Den Borre, Tanguy Segers, Soheil Ashkani-Esfahani, Daniel Guss, Cesar De Cesar Netto, Christopher W. DiGiovanni, Jan Victor, Emmanuel Audenaert, Arne Burssens

**Affiliations:** 1 Resident Orthopaedic Surgery, Department of Orthopaedics and Traumatology, Ghent University Hospital, Corneel Heymanslaan 10, OVL, 9000 Gent, Belgium; 2 Department of Human Structure and Repair, Ghent University, Corneel Heymanslaan 10, OVL, 9000 Ghent, Belgium; 3 Foot and Ankle Research and Innovation Laboratory (FARIL), Harvard Medical School, Massachusetts General Hospital, Boston, MA USA; 4 Department of Orthopaedics, Duke University, Durham, North Carolina USA; 5 Department of Orthopaedics and Traumatology, Ghent University Hospital, Corneel Heymanslaan 10, OVL, 9000 Gent, Belgium; 6 Department of Trauma and Orthopedics, Addenbrooke’s Hospital, Cambridge University Hospitals NHS Foundation Trust, Hills Road, Cambridge, CB2 0QQ UK; 7 Department of Electromechanics, Op3Mech Research Group, University of Antwerp, 2020 Antwerp, Belgium

**Keywords:** Computer science, Bone, Biomedical engineering

## Abstract

Detection of syndesmotic ankle instability remains challenging in clinical practice due to the limitations of two-dimensional (2D) measurements. The transition to automated three-dimensional (3D) measurement techniques is on the verge of a breakthrough but normative and side-to-side comparative data are missing. Therefore, our study aim was two-fold: (1) to establish 3D anatomical reference values of the ankle syndesmosis based on automated measurements and (2) to determine to what extent the ankle syndesmosis is symmetric across all 3D measurements. Patients without syndesmotic pathology with a non-weight-bearing CT scan (NWBCT; N = 38; Age = 51.6 ± 17.43 years) and weight-bearing CT scan (WBCT; N = 43; Age = 48.9 ± 14.3 years) were retrospectively included. After training and validation of a neural network to automate the segmentation of 3D ankle models, an iterative closest point registration was performed to superimpose the left on the right ankle. Subsequently, 3D measurements were manually and automatically computed using a custom-made algorithm and side-to-side comparison of these landmarks allowed one to investigate symmetry. Intra-observer analysis showed excellent agreements for all manual measurements (ICC range 0.85–0.99) and good (i.e. < 2.7° for the angles and < 0.5 mm for the distances) accuracy was found between the automated and manual measurements. A mean Dice coefficient of 0.99 was found for the automated segmentation framework. The established mean, standard deviation and range were provided for each 3D measurement. From these data, reference values were derived to differ physiological from pathological syndesmotic alignment. Furthermore, side-to-side symmetry was revealed when comparing left to right measurements (P > 0.05). In clinical practice, our novel algorithm could surmount the current limitations of manual 2D measurements and distinguish patients with a syndesmotic ankle lesion from normal variance.

## Introduction

Syndesmotic ankle injuries are present in up to 18% of all ankle sprains and up to 20% of ankle fractures requiring internal fixation^1^. If undiagnosed and therefore left untreated, they may lead to syndesmotic instability in the short-term and subsequent posttraumatic ankle osteoarthrtis in the long-term^2–4^. Diagnosing syndesmotic instability can be challenging, especially when subtle^5^. The current diagnostic work-up involves manual measurements of the injured distal tibiofibular joint on plain radiographs in comparison to the non-injured side^6^. However, discordance exist in the current literature whether the configuration of the ankle syndesmosis is symmetrical^7,8^. Moreover, certain types of displacement such as rotation within the distal tibiofibular joint cannot be assessed on plain radiography due to superposition of the osseous structures^9,10^. Conventional CT imaging overcomes this shortcoming and allows different angular measurements to quantify rotation^11^. The recent advent of weightbearing CT (WBCT) improves upon conventional non-weight-bearing CT (NWBCT) by allowing a bilateral assessment of the ankle syndesmosis during physiological stance^12–15^. Using WBCT, novel noninvasive three-dimensional imaging methods have been deployed to accurately identify patients with a history of syndesmotic ankle injuries^16,17^. Currently, various methods have been reported to compare syndesmotic alignment, mostly by use of manual measurements on 2D axial slices^18^. These 2D metrics, however, correlate poorly with the actual 3D deviation of the fibula require a time-consuming endeavor while some measurements are subject to intra- and interobserver inaccuracies^19–21^. Current syndesmotic volume measurement techniques are also cumbersome to employ and not integrated into the viewing programs commonly used in today’s clinical practice^22,23^. These drawbacks indicate that an accurate diagnosis of syndesmotic lesions should involve a—preferably automated—3D analysis^24,25^. In this study, we aim to bridge this gap by presenting a computational algorithm for automated 3D assessment of the distal tibiofibular joint. Specifically, measurements will be performed on 3D reconstructed bony models rather than only on selected 2D image slices, revealing the syndesmotic alignment in all 6 degrees of freedom. Moreover, these measurements will be performed by an automated computational algorithm, avoiding human error of manual measurements^20^.

Since this study presents a novel approach, 3D reference values are not yet established for the normal ankle syndesmosis, nor is it clear to what extent the 3D configuration of the normal ankle syndesmosis is symmetrical during non- or weightbearing conditions. Therefore, the objectives of this study are (1) to determine physiological reference values concerning the 3D configuration of the normal ankle syndesmosis based on an automated method, (2) to determine whether the ankle syndesmosis is symmetric across all 3D measurements on both weightbearing and non-weightbearing imaging. In this respect, we hypothesize that (1) broad reference ranges will be present due to inter-individual differences and (2) side to side differences will be present in the normal ankle syndesmosis.

## Material and methods

### Study population and design

**Figure 1:**
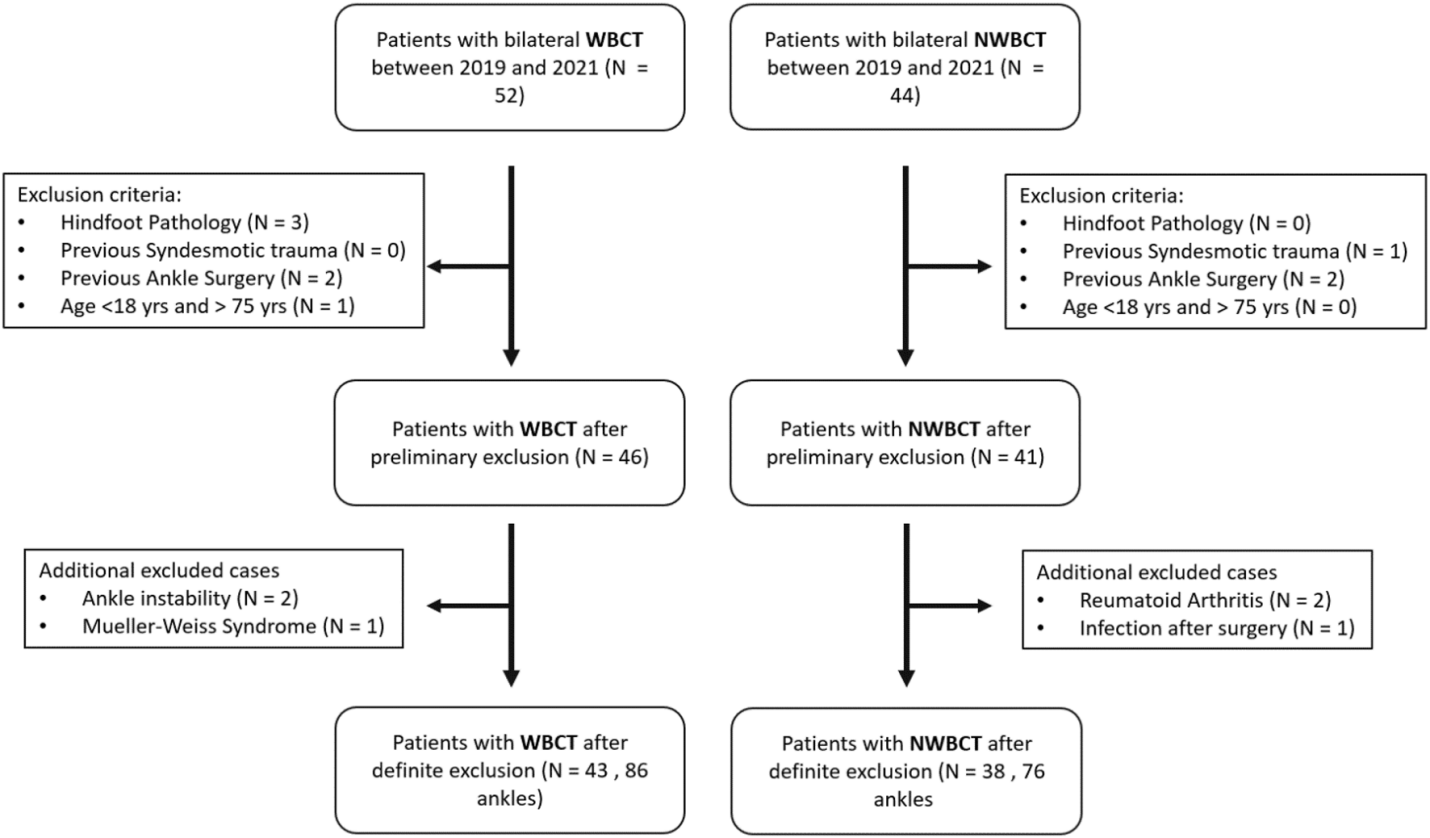
Patient selection flowchart with exclusion criteria.

In this retrospective comparative cohort study, patients with a NWBCT (N = 38; Mean age = 51.61 years, SD = 17.43) and WBCT (N = 43; Mean age = 48.93 years; SD = 14.35) were analyzed. Inclusion criteria were a bilateral NWBCT and/or WBCT of the foot and ankle between January 2019 and December 2021. Exclusion criteria consisted of hindfoot pathology, previous traumatic injuries to the ankle syndesmosis, previous ankle surgery and an age less than 18 years or more than 75 years. In Fig. 1, a detailed overview of the patient selection process is depicted. The study was conducted in accordance with the Declaration of Helsinki and the Guidelines for Good Clinical Practice. The Institutional Review Board of the University Hospital of Ghent approved this study (OG10601102015) and informed consent was waived.

A bilateral non-WBCT was carried out with the patient positioned supine in a Siemens SOMATOM (Siemens Medical Solutions, Munich, Germany) CT scan. Following imaging protocol and settings were used for the non-WBCT: tube voltage, 140 kV; tube current, 156 mAs; CTDIvol 16.07 mGy; matrix, 512 512; the pixel size varied according to the topogram slice interval (0.6 mm). The non-WBCT was performed while the ankle was in a 90 degree plantarflexion position, as used in standard clinical practice. Bilateral WBCT images were acquired by a PedCAT device (Curvebeam, Hatfield, PA, USA) in which patients were positioned with both feet parallel at shoulder width. The PedCAT was used at the following settings: tube voltage: 96 kV; tube current: 7.5 mAs; CTDIvol: 4.3 mGy; matrix: 160 × 160 × 130; pixel size: 0.4 mm; slice interval: 0.4 mm^26^.

### 3D analysis

#### Automated segmentation framework

The initial process of segmenting the CT images into 3D structures was performed semi-automatically. CT images were imported in Materialise’s Interactive Medical Image Control System (Mimics^®^ v21.0, Materialise, Leuven, Belgium) using a Digital Imaging and Communications in Medicine (DICOM) format. In Mimics^®^, the distal tibia and distal fibula were segmented semi-automatically to form 3D stereolithography (STL) volumes, composed of a number of vertices and faces. While the WBCT scans covered the tibia and fibula until approximately half of its lengths, the non-WBCT scans covered the whole tibia. In the latter, only the distal half of the tibia and fibula were segmented to have equivalent lengths on the WBCT and non-WBCT scans.

**Figure 2:**
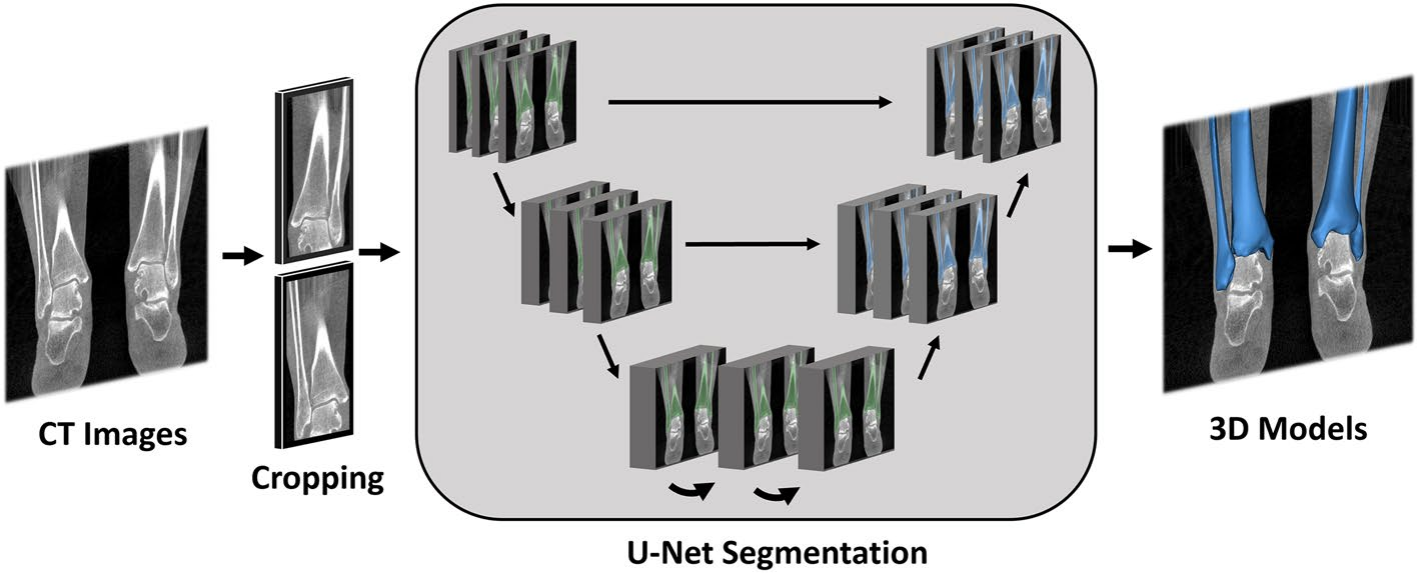
Deep learning framework. CT images were cropped to the region of interest, after which a 3D U-Net network was trained for automated tibia and fibula segmentation. After that, the segmentation masks were converted into 3D bony models. Created with Mimics® (version 21.0, Materialise, Leuven, Belgium, https://www.materialise.com/en/healthcare/mimics-innovation-suite/mimics).

In order to establish a fully automated algorithm, a neural network was trained to automate the segmentation step using artificial intelligence. We employed a standard 3D U-NET architecture from the MONAI framework^27^. The training and validation of the multiclass model were conducted using a fivefold cross-validation procedure on the cropped CT volumes (Fig. 2). In each fold, the network underwent 200 epochs of training. During the training process, the network parameters were updated using an ADAM optimizer in order to minimize the cross-entropy loss function, with a learning rate set to 10^(−3)^. To evaluate the performance of the model, we utilized the dice similarity metric, which was calculated for the five distinct test folds. The dice metric provides a quantitative measure of the overlap between the predicted and ground truth segmentation masks, serving as an assessment of the model's accuracy^28^.

**Figure 3:**
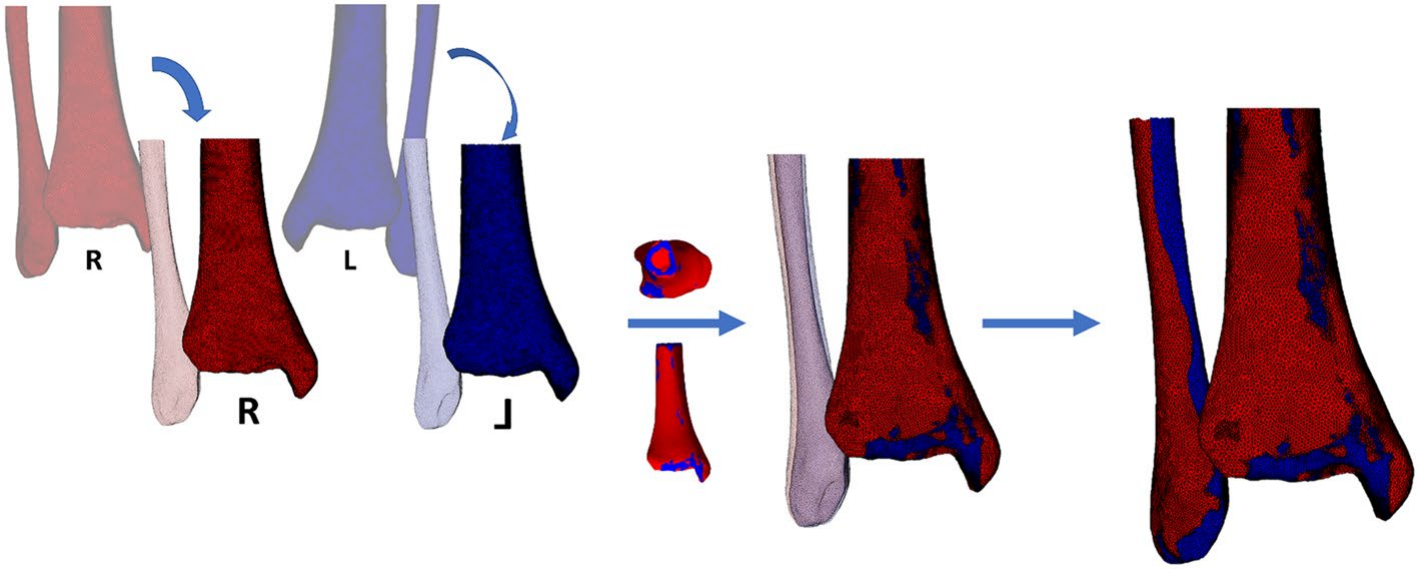
Mirroring and alignment of the right (red) and left (blue) ankle. The left ankle tibia was mirrored and rigidly registered to the right tibia. The Procrustes transformation matrix of each left tibia was subsequently deployed on the corresponding left fibula, in order to retain the subject-specific anatomical configuration of the ankle syndesmosis. Created with Matlab^®^ (version R2020b, MathWorks, Natick, MA, USA, https://www.mathworks.com/products/matlab.html).

After segmentation, STL’s were imported in a custom-made script in Matlab^®^ software (R2020b, MathWorks, Natick, MA, USA). Using this script, STL models were firstly geodesically re-meshed to a surface mesh consisting of homogenous triangular surfaces with a mean edge length of 2 mm. Subsequently, the left tibia was mirrored and rigidly registered to the right tibia using an iterative closest point (ICP) algorithm. The Procrustes transformation matrix of each left tibia was subsequently deployed on the corresponding fibula, in order to retain the subject-specific anatomical configuration of the ankle syndesmosis (Fig. 3).

#### Automated 3D landmark detection

**Figure 4:**
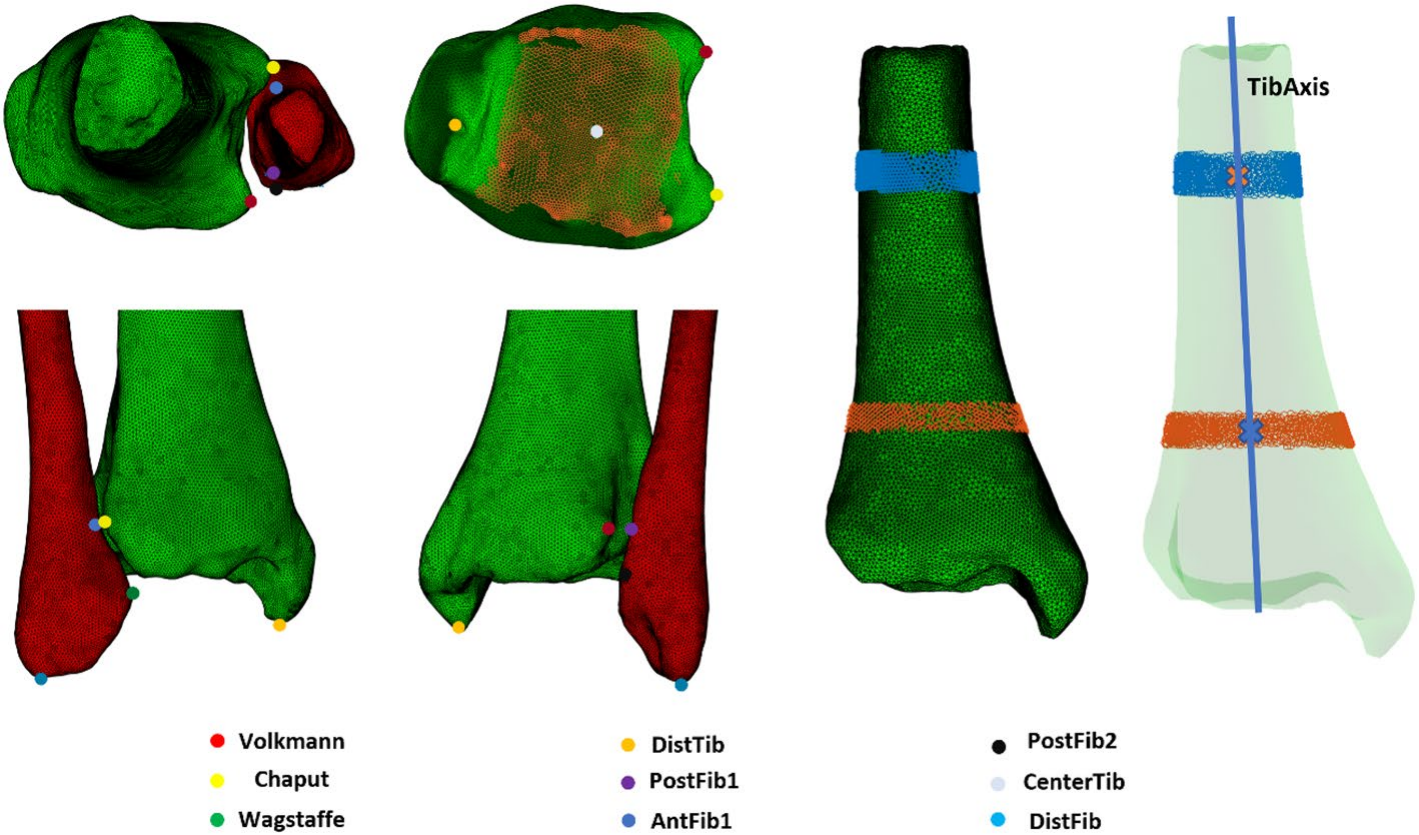
Anatomical landmarks and axes of the ankle syndesmosis, automatically derived by the algorithm. DistTib (yellow), Most distal point of the tibia on the medial malleolus. DistFib (light blue), Most distal point of the lateral malleolus. CenterTib (light grey), Center of the tibial plafond (orange dots). AntFib1 (dark blue), geometrical nearest point on the fibula to the Chaput tubercle. PostFib1 (purple), geometrical nearest point on the fibula to the Volkmann tubercle. PostFib2 (black), posterior fibular tubercle. TibAxis (blue), anatomical tibia axis. Created with Matlab^®^ (version R2020b, MathWorks, Natick, MA, USA, https://www.mathworks.com/products/matlab.html).

Several anatomical axes and landmarks were automatically determined based on geometrical information (Fig. 4, Table 1). Global and local constraints for each landmark were extracted by considering relative positions of these landmarks and differential geometric features.

**Table 1:** 3D Anatomical landmarks of the ankle syndesmosis with their respective method of calculation.

Landmark	Method of calculation
SupTib	Geometrical center of the vertices of the 75th and 80th quantile of the tibia
InfTib	Geometrical center of the vertices of the 45th and 50th quantile of the tibia
Chaput	Most lateral coordinate of the anterior half of the tibia
Volkmann	Most lateral coordinate of the posterior half of the tibia
DistTib	Most inferior coordinate along the anatomical tibia axis
CenterTib	Geometrical center of the tibial plafond
DistFib	Most inferior coordinate along the anatomical fibula axis
AntFib_1_	Nearest geometrical neighbouring coordinate of the Chaput tubercle on the fibula
PostFib_1_	Nearest geometrical neighbouring coordinate of the Chaput tubercle on the fibula
Wagstaffe	Most anterior vertex along the second principal component axis of the fibula
PostFib_2_	Most posterior point along the anteroposterior axis of the ankle

**Figure 5:**
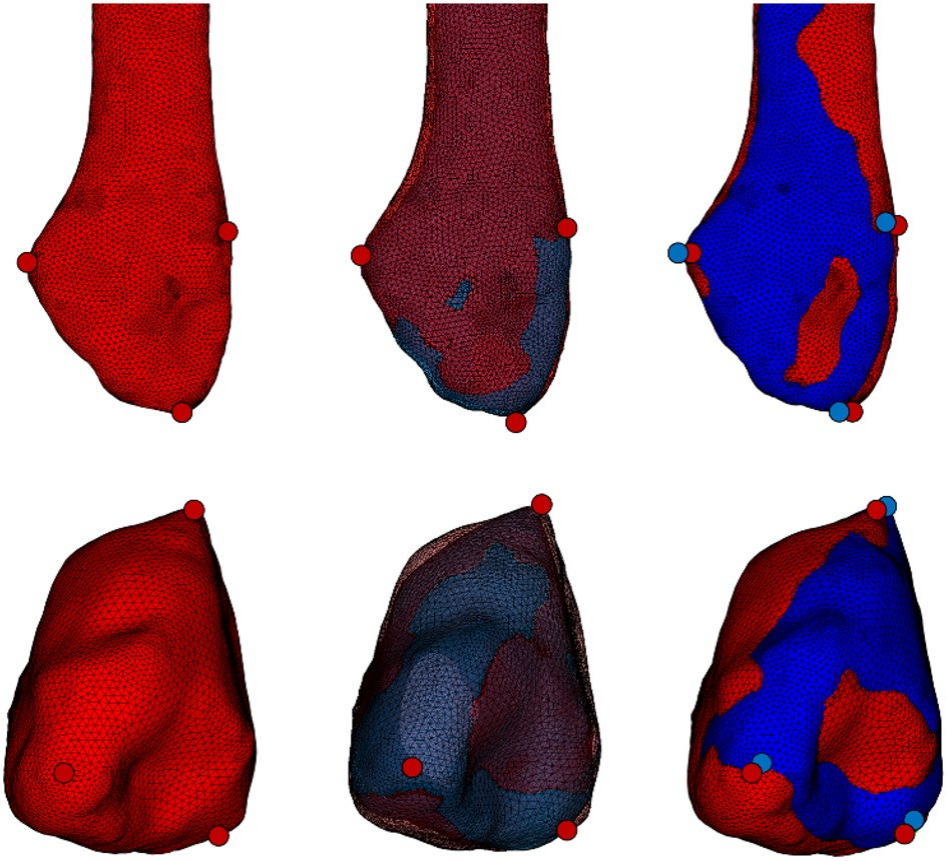
Landmark transfer from the right (red) to the left (blue) fibula. Left, The landmarks were firstly computed on the right fibula. Center, The right and left fibula were rigidly registered based on iterative closest point analysis. Landmarks were transferred from the right fibula to the left fibula by a nearest neighbor algorithm. Right, the inverse transformation matrix was applied to the left fibula to reposition it to its original location. Created with Matlab^®^ (version R2020b, MathWorks, Natick, MA, USA, https://www.mathworks.com/products/matlab.html).

Landmarks were subsequently detected by combining these constraints. The anatomical axis of the distal tibia was determined by connecting 2 points. The first point (SupTib) was represented by the geometrical center of the vertices containing the 75th and 80th quantile of the tibia along the supero-inferior axis. The second point (InfTib) was represented by the geometrical mean of the vertices containing the 40th and 45th quantile^29^. The latter was chosen considering this quantile was positioned superiorly to the medial malleolus, which could otherwise affect the axis in a medial direction. The anatomical axis of the fibula was calculated based on principal component analysis (PCA) of the three-dimensional coordinates of the vertices, based on Carrara et al.^30^ Following, several anatomical landmarks were computationally derived based on these axes. The most distal point of the tibia (DistTib) was identified by calculating the most inferior point along the anatomical tibia axis. Similarly, the most distal point of the right fibula (DistFib) was identified, by calculating the most inferior point along the anatomical fibula axis. The center of the tibial plafond (CenterTib) was identified by calculating the geometrical mean coordinate of the vertices representing the tibial plafond. This was performed using the following methodology: the tibia was first aligned along its anatomical axis. We then determined the intersection point of this axis with the lower part of the tibia, which served as the 'baseline point' on the tibial plafond. From this baseline point, we defined a region of interest encompassing the tibial plafond. This region included all the vertices that were located within 5 mm below and 5 mm above the baseline point. Next, we calculated the normals of these vertices, which are vectors perpendicular to the surface. We only retained the vertices whose normals pointed downward in the Z-direction, with a Z-component lower than − 0.8. These selected vertices represented the tibial plafond surface. Finally, we computed the center vertex of these selected points to identify the central point of the tibial plafond. Next, the anterior (*Chaput*) and posterior (*Volkmann*) tibial tubercle point on the incisura were identified^31^, by calculating the most extreme lateral vertex coordinate of the tibia along the transmalleolar axis of both the anterior and posterior half of the tibia, respectively. The geometrical nearest neighboring coordinate (in the 3D space) on the fibula from the *Chaput* and *Volkmann* tubercle were computationally derived, representing respectively AntFib_1_ and PostFib_1_. Thereafter, the anterior (*Wagstaffe*)^31^ fibular tubercle was computationally derived, by calculating the most extreme anterior vertex coordinate along the second principal component axis of the distal fibula. The posterior fibular tubercle (PostFib_2_) was calculated as most posterior point along the anteroposterior axis of the ankle. In order to derive identical landmarks on the right and left fibula, the landmarks were firstly computed on the right fibula. Thereafter, the right and left fibula were rigidly registered using ICP algorithm. Landmarks were transferred from the right fibula to the left fibula by a nearest neighbor algorithm. Finally, the inverse transformation matrix was applied to the left fibula to reposition it to its original location (Fig. 5).

#### Automated measurements

**Figure 6:**
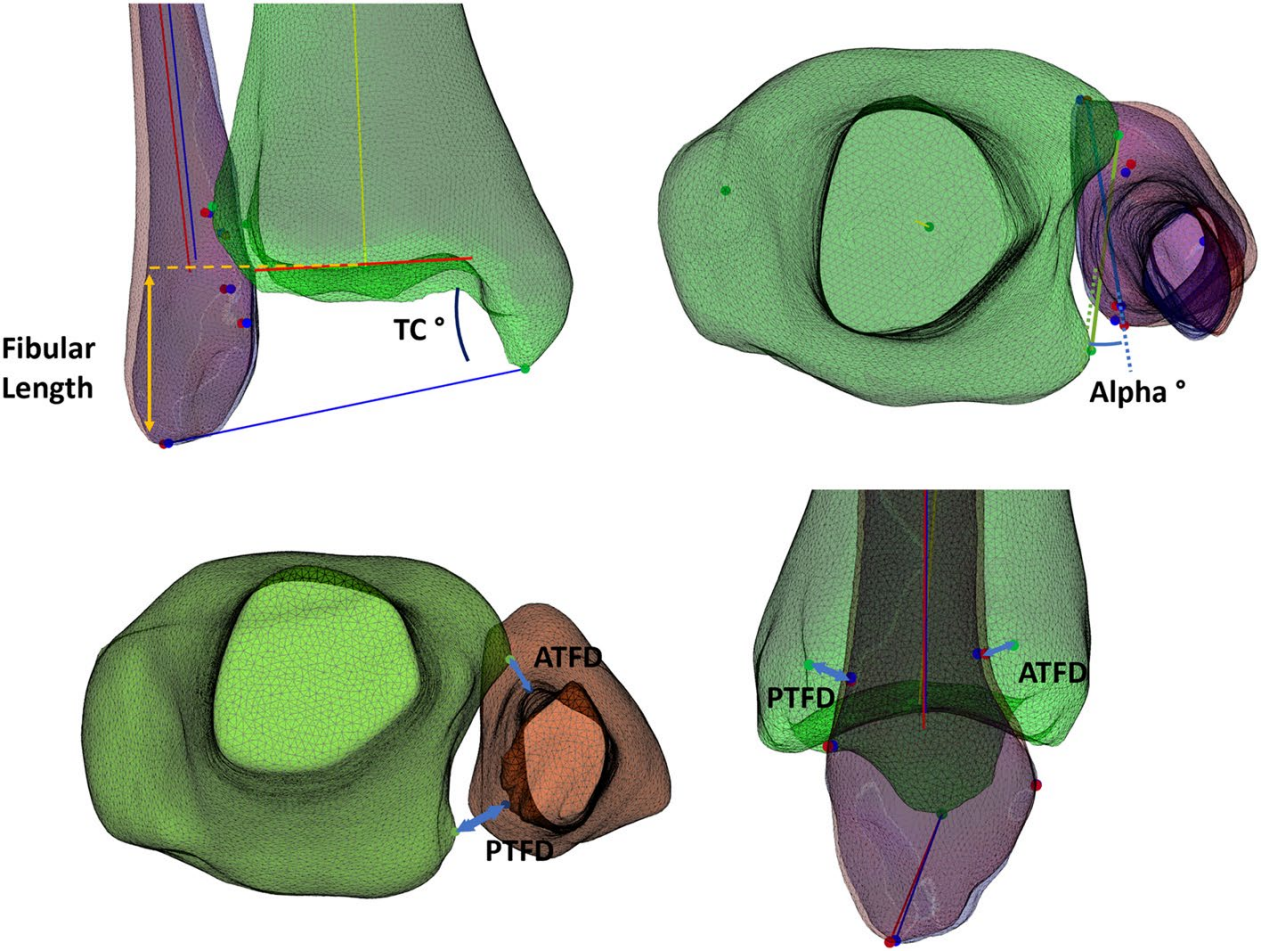
Automated measurements of the distal tibiofibular syndesmosis. Left, The Fibular Length was calculated as the distance between the center of the tibial joint line and DistFib, in the coronal plane. The Talocrural Angle was represented as an angle between the tibial articular surface line and a line connecting TibDist to FibDist. Middle, The Alpha angle represented the angle between a line connecting Chaput to Volkmann tubercle and a line connecting AntFib1 to PostFib1, in the axial plane. Right, The Anterior TibioFibular Distance (ATFD) represented the distance between the Chaput tubercle and AntFib1. Similarly, the Posterior TibioFibular Distance (PTFD) represented the 3D distance between the Volkmann tubercle and PostFib1. Created with Matlab^®^ (version R2020b, MathWorks, Natick, MA, USA, https://www.mathworks.com/products/matlab.html).

Based on these anatomical landmarks, the following measurements were automatically calculated in the Matlab^®^ script (Table 2); The Anterior TibioFibular Distance (ATFD), Posterior TibioFibular Distance (PTFD), Alpha angle^32^, Fibular Length and Talocrural Angle. These measurements are explained in further detail in Fig. 6. To investigate the reliability of these measurements, all these landmarks were also identified and marked by a senior orthopaedic resident (MP), 2 weeks apart. Measurements were then performed on these manually marked landmarks to investigate both the intra-operator variability and accuracy of the automated measurements.

**Table 2:** The measurements defining the alignment of the syndesmosis, their description and clinical significance.

Measurement	Description	Clinical significance
ATFD	Distance between Chaput tubercle and AntFib_1_	Anterior diastasis of the distal tibiofibular joint
PTFD	Distance between Volkmann tubercle and PostFib_1_	Posterior diastasis of the distal tibiofibular joint
Alpha angle	Angle between a line connecting Chaput to Volkmann tubercle and a line connecting AntFib_1_ to PostFib_1_ (negative value = internal rotation)	Rotation of the fibula (increase = external rotation of the fibula)
Fibular length	Distance between CenterTib and DistFib in the coronal plane	Shortening of the fibula
Talocrural angle	Angle between the tibial articular surface line and a line connecting TibDist to FibDist in the coronal plane	Shortening of the fibula (decrease = shortening)

### Statistical analysis

A priori power analysis was performed with G*Power (Version 3.1.9.2, Dusseldorf University, Dusseldorf, Germany). The effect size was obtained from previous studies^]^. Calculations have shown that a total sample size of 8 needs to be achieved when comparing 2 groups for the calculated effect size (f = 1.84) with a power level of 0.95 and a level of significance set at 0.05. Intra-class correlation coefficient (ICC) was used to evaluate intra-observer measurement differences of the manual 3D measurements. The mean error between the automated and manual measurements were calculated for each variable.

A Shapiro–Wilk test showed that the data were normally distributed. Subsequently, a Levene’s revealed homogeneity of variances among the studied groups. Based on the outcome of these tests, we determined that a Paired, two-tailed, Student’s t-test between all measurements could be used to compare whether side-to-side differences existed. Descriptive statistics (Mean, Standard Deviation (SD) and Range) were calculated for each of the continuous study variables. Reference ranges were defined based on similar studies^8,35,36^, distinguishing abnormal values as localized outside two standard deviations from the mean. The SPSS (release 28.0.0. standard version, SPSS, Inc., Chicago, IL, USA) statistical package was used to analyze the results. A probability level of *P* < 0.05 was considered significant.

## Results

**Figure 7:**
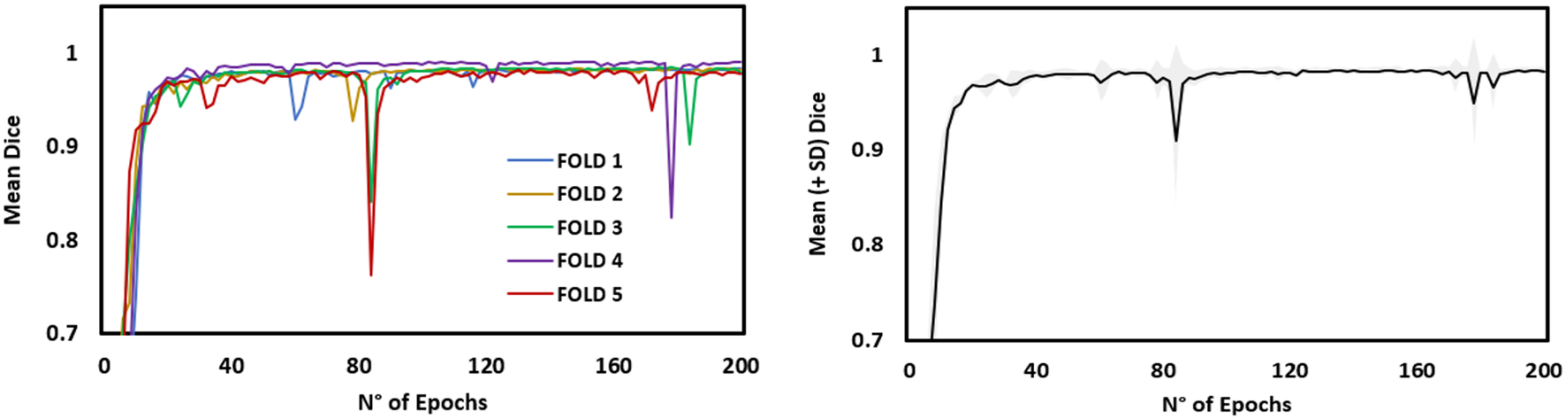
3D U-Net segmentation accuracy. Left, evolution of the Dice coefficient for all five folds. Right, mean (+ SD) evolution of the Dice coefficient. *SD* standard deviation.

For the automated U-Net segmentation, a mean dice coefficient of 0.99 (SD 0.003) was calculated for the five folds, representing excellent accuracy (Fig. 7). ICC analysis between manual observations showed excellent agreement with values of 0.90, 0.89, 0.85, 0.99 and 0.89 when measuring the Alpha angle, ATFD, PTFD, Fibular Length and Talocrural angle, respectively^37^. A mean error of 2.63° (SD 2.58), 0.26 mm (SD 0.28), 0.43 mm (SD 0.59), 0.25 mm (SD 0.37) and 2.49° (SD 2.06) was found between the automated and manual measurements for the Alpha angle, ATFD, PTFD, Fibular Length and Talocrural angle, respectively. The mean, SD, range and derived normative data within two standard deviations are given for each measurement (Table 3). No statistically significant differences could be detected between 3D measurements of left and right ankles for all variables (Table 4).

**Table 3:** Mean (Standard Deviation), Range and Derived Reference Value (based on two standard deviations from the mean) for the automated measurements.

Measurement	Mean (SD)		Range		Derived reference value	
	NWBCT	WBCT	NWBCT	WBCT	NWBCT	WBCT
ATFD	2.94 (1.19)	2.78 (1.05)	[0.22; 6.64]	[0.52; 6.30]	[0.61; 6.27]	[0.72; 4.93]
PTFD	4.85 (1.64)	4.91 (1.18)	[1.00; 8.72]	[2.64; 8.11]	[1.63; 8.06]	[2.60; 7.23]
Alpha angle	19.01 (6.02)	20.10 (7.27)	[3.18; 32.96]	[3.98; 33.53]	[7.22; 30.80]	[5.85; 34.35]
Fibular length	25.14 (3.62)	25.56 (3.21)	[16.09; 32.27]	[19.57; 34.04]	[18.05; 32.24]	[19.28; 31.85]
Talocrural angle	17.81 (4.67)	14.79 (3.96)	[7.61; 25.82]	[9.04; 28.99]	[8.66; 29.96]	[7.02; 22.56]

*WBCT* weightbearing CT, *NWBCT* non-weightbearing CT.

**Table 4:** Mean (Standard Deviation) values of the right and left syndesmotic measurements, with their respective difference and p-value.

Measurement	Mean (SD)						p-value	
	Right		Left		Difference			
	NWBCT	WBCT	NWBCT	WBCT	NWBCT	WBCT	NWBCT	WBCT
ATFD	2.92 (0.86)	2.73 (0.93)	2.95 (1.44)	2.83 (1.15)	0.79	0.58	0.06 (NS)	0.59 (NS)
PTFD	4.71 (1.48)	4.95 (1.05)	4.98 (1.78)	4.88 (1.30)	0.95	0.56	0.88 (NS)	0.41 (NS)
Alpha angle	19.73 (5.60)	20.43 (6.87)	18.28 (6.32)	20.22 (7.65)	3.40	1.99	0.20 (NS)	0.56 (NS)
Fibular length	25.08 (3.44)	25.50 (3.25)	25.21 (3.79)	25.62 (3.17)	0.72	0.56	0.62 (NS)	0.34 (NS)
Talocrural angle	17.74 (4.68)	14.52 (3.93)	17.88 (4.66)	14.65 (4.00)	0.88	0.67	0.62 (NS)	0.34 (NS)

*WBCT* weightbearing CT, *NWBCT* non-weightbearing CT, *NS* non-significant.

## Discussion

Contralateral radiographic comparison after ankle trauma is frequently performed using manual measurements to rule out a syndesmotic injury. While semi-automated distance or volume measurement techniques are emerging^16,19,22,23^, they are still performed on distinct 2D CT image slices. Therefore, this study presented an automated algorithm to perform measurements on 3D models of the ankle syndesmosis using a cohort without ankle pathology, imaged by non-weightbearing and weightbearing CT. In doing so, we aimed to establish normative data on the 3D alignment and symmetry of the ankle syndesmosis. Furthermore, we provided distinct reference values to differ physiological from pathological syndesmotic alignment, while side-to-side symmetry was revealed when comparing left to right measurements.

Based on the normative data, an ATFD value greater than 4.93 mm and 6.27 mm on WBCT and NWBCT, respectively, could be indicative for pathological anterior widening of the DTFJ. A PTFD value greater than 7.23 mm and 8.06 mm on WBCT and NWBCT, respectively, could be indicative for pathological posterior widening of the DTFJ. An alpha angle smaller than 5.85° and 7.22°, on WBCT and NWBCT respectively, could be indicative for pathological external rotation of the distal fibula within the incisura. A talocrural angle smaller than 7.02 mm and 8.66 mm in conjunction with a fibular length smaller than 19.28 mm and 18.05 mm on WBCT and NWBCT, respectively, is indicative for shortening of the distal fibula.

Previous studies examined the normal ankle syndesmosis using measurements on 2D CT images^11,32,38,39^. Two studies provide reference values based on non-weightbearing CT describing the distal tibiofibular distance and rotation^11,38^. However, those data were obtained from unilateral CT scans and did not assess the syndesmosis for symmetry. This flaw was surmounted by pioneering studies performing side-to-side comparisons of the normal ankle syndesmosis using weightbearing CT^32,39^. Based on these important results, Hagemijer et al.^39^, concluded that the contralateral non-injured ankle could serve as a valid internal control to detect syndesmotic ankle injury. Our results parallel these findings by demonstrating a 3D symmetry of the normal ankle syndesmosis. These findings strengthen the recommendation to use the opposite ankle as a comparison when using CT imaging. However, having such a reference range remains useful in cases where bilateral injuries are suspected or no data for comparison is available (i.e., unilateral CT scans).

Vetter et al. have additionally described superior reliability to measure the fibular rotation on axial CT slices 6 mm below the talar joint level^40^. Despite the relevance of these results, technical advances are now emerging in the direction of three-dimensional models and anatomical landmarks to better understand foot and ankle disorders, not relying on selected CT slices^16^. Currently, 3D volume-based measurements are frequently used to detect syndesmotic ankle injuries^22,23^. These studies found a higher sensitivity compared to conventional measurements and the obtained volume corresponded with the amount of syndesmotic ligament injuries. However, it remains difficult to describe the direction of displacement and rotation, because this requires coordinates of anatomical landmarks relative to a fixed reference frame^16,17^. To the best of our knowledge, our results go beyond these previous reports by providing the first reference values of measurements of the ankle syndesmosis on 3D reconstructed bony models, calculated using an automated computational algorithm. Automatization of the analysis greatly reduces the burden of performing manual measurements. In clinical practice, these can be applied to detect acute syndesmotic ankle injuries and potentially aid in the reconstruction of chronic instability by correcting the amount of calculated deviation relative to uninjured side, e.g. in case of a malrotated or shortened fibula^41^.

The present study contains several limitations. First, the study was performed on a retrospective imaging database. While all patients’ charts were reviewed to exclude previous ankle trauma and degeneration of the distal tibiofibular joint, clinical presence of ankle instability could not be ruled out. This condition might impact the congruency on the ankle syndesmosis, because of the overlap demonstrated between injuries to the syndesmotic and lateral ligaments of the ankle^42^. In addition, the patients in the study cohorts were referred for foot ankle disorders albeit the ankle, which make them presumably not completely representative for the general population. However all previous studies describing reference values based on bilateral weightbearing CT imaging, involved study cohorts imaged for similar indications. Nevertheless, future studies could be improved by including healthy individuals without a history of foot and ankle complaints. Secondly, the cohort who sustained non-weightbearing CT imaging was different from the cohort imaged by weight bearing CT. Still, it can be considered of relevance to know whether reference data on the normal ankle syndesmosis differ, when obtained from a cohort imaged by either non-weightbearing or weightbearing CT. Third, congruency of the ankle syndesmosis depends on the osseous configuration and the condition of the ankle syndesmotic ligaments. Measurements on the osseous configuration could be well derived from the CT scans in the present study, but no data on the condition of the syndesmotic ligaments could be collected. For this reason, additional MR imaging could be an added value in future studies, because of the high accuracy to detect disruption of syndesmotic ankle ligaments^43^. Fourthly, we did not investigate potential differences in the syndesmotic shape and how this may affect our findings. Previous studies have demonstrated that there exist significant variability in the morphology of the tibial incisura, which should be taken into account when quantifying the syndesmotic configuration^7,44–46^ Lastly, while cutoffs were defined for abnormal values of the presented variables based on normative data, these were not correlated to measurements in patients with syndesmotic injuries. Future case–control studies should look into the difference of these variables between healthy controls and patients with syndesmotic injuries, including a larger sample size, to validate these cutoff values. Furthermore, future studies should look at these reference values while using recently described CT scans with augmented external rotation stress to detect subtle syndesmotic instability, and should also look into the potential segmentation error when using semi-automated segmentation software^47^.

## Conclusion

In this study, we have established a novel automated algorithm to assess the 3D syndesmotic alignment. Subsequently, we have used this algorithm to define normative reference values, revealing a side-to-side symmetry. This allows to capture the alignment of the ankle syndesmosis in all six degrees of freedom, improving upon previously established measurements on 2D plain radiographs or CT image slices. In clinical practice, this 3D analysis and accompanying reference values could facilitate the differentiation between syndesmotic ankle lesions and normal variance. Future research could improve the present study by using prospectively recruited cohorts with additional clinical and MRI investigations to assure an intact condition of the syndesmotic ligaments. It could also be assessed if these 3D techniques could aid in the treatment of syndesmotic ankle injuries by correcting the amount of calculated deviation relative to uninjured side, e.g. in case of a malrotated or shortened fibula.

## Data Availability

The datasets used and/or analysed during the current study are available from the corresponding author on reasonable request.
